# Expediting systematic reviews: methods and implications of rapid reviews

**DOI:** 10.1186/1748-5908-5-56

**Published:** 2010-07-19

**Authors:** Rebecca Ganann, Donna Ciliska, Helen Thomas

**Affiliations:** 1 School of Nursing, Faculty of Health Sciences, McMaster University, Hamilton, Canada

## Abstract

**Background:**

Policy makers and others often require synthesis of knowledge in an area within six months or less. Traditional systematic reviews typically take at least 12 months to conduct. Rapid reviews streamline traditional systematic review methods in order to synthesize evidence within a shortened timeframe. There is great variation in the process of conducting rapid reviews. This review sought to examine methods used for rapid reviews, as well as implications of methodological streamlining in terms of rigour, bias, and results.

**Methods:**

A comprehensive search strategy--including five electronic databases, grey literature, hand searching of relevant journals, and contacting key informants--was undertaken. All titles and abstracts (n = 1,989) were reviewed independently by two reviewers. Relevance criteria included articles published between 1995 and 2009 about conducting rapid reviews or addressing comparisons of rapid reviews versus traditional reviews. Full articles were retrieved for any titles deemed relevant by either reviewer (n = 70). Data were extracted from all relevant methodological articles (n = 45) and from exemplars of rapid review methods (n = 25).

**Results:**

Rapid reviews varied from three weeks to six months; various methods for speeding up the process were employed. Some limited searching by years, databases, language, and sources beyond electronic searches. Several employed one reviewer for title and abstract reviewing, full text review, methodological quality assessment, and/or data extraction phases. Within rapid review studies, accelerating the data extraction process may lead to missing some relevant information. Biases may be introduced due to shortened timeframes for literature searching, article retrieval, and appraisal.

**Conclusions:**

This review examined the continuum between diverse rapid review methods and traditional systematic reviews. It also examines potential implications of streamlined review methods. More of these rapid reviews need to be published in the peer-reviewed literature with an emphasis on articulating methods employed. While one consistent methodological approach may not be optimal or appropriate, it is important that researchers undertaking reviews within the rapid to systematic continuum provide detailed descriptions of methods used and discuss the implications of their chosen methods in terms of potential bias introduced. Further research comparing full systematic reviews with rapid reviews will enhance understanding of the limitations of these methods.

## Background

Healthcare increasingly demands rapid access to current research to ensure evidence-informed decision making and practice. Emerging issues require access to high-quality evidence in a timely manner to inform system and policy response. In addition, government decision-makers request evidence to be delivered in shortened timeframes. Rapid reviews are literature reviews that use methods to accelerate or streamline traditional systematic review processes. Target audiences for rapid reviews include government policymakers, healthcare institutions, health professionals, and patient associations to inform health system planning and policy development [1,2]. Conclusions often focus on federal, regional, or local jurisdictional contexts; some recommendations caution readers about limitations in transferability to other jurisdictions or contexts [1,3]. Rapid review methodologies may be driven by clinical urgency and intense demands for uptake of technology, or may be determined by limited time and resources to conduct full systematic reviews [4].

Systematic reviews typically take a minimum of six months to one year to complete. To address requests for literature reviews in shorter time periods (*e.g*., one to six months), and to facilitate informed decision making, it is imperative to understand the various rapid review strategies. There is little empirical evidence comparing the continuum of products among rapid reviews and full systematic reviews, or analysing the diverse methods used in rapid reviews [5]. It is important not only to establish transparent methodologies for rapid reviews, but also to understand the implications of what is lost in terms of rigour, bias, and results when methods associated with full systematic review are streamlined.

## Objectives

1. What are the methods used for rapid review?

2. Are there any comparisons of rapid versus traditional review methods for the same topic?

3. What are the implications of taking methodological shortcuts from a traditional Cochrane review? What biases increase?

## Methods

### Search strategy

A systematic search was conducted in February 2008 of MEDLINE (1996 to October Week 1 2009), CINAHL (1982 to October week 1 2009), PsychInfo (1985 to October week 1 2009) and EMBASE (1996 to 2009 week 40). Search terms used in the databases included: 'realis* revew.mp.' [mp = title, original title, abstract, name of substance word, subject heading word], 'realis* synthesis.mp.,' 'realis* evaluation.mp,' '(meta-method or meta method),' 'realis* approach.mp,' '(meta-evaluation or meta evaluation),' ((rapid literature review) or (rapid systematic review) or (rapid scoping review)) or ((rapid review) or (rapid approach) or (rapid synthesis)) or ((meta-method* or meta method*) or (meta-evaluation* or meta evaluation*) or (rapid evidence assess*)). The database searches were limited to English language articles dated between 1996 and 2009.

The Scholars' Portal database was searched using the terms: KW = (realis* review or (realis* approach) or (realis* synthesis)) or KW = ((realis* approach) or meta-method or (meta method)) or KW = (meta-evaluation or (meta evaluation)) ((rapid literature review) or (rapid systematic review) or (rapid scoping review)) or ((rapid review) or (rapid approach) or (rapid synthesis)) or ((meta-method* or meta method*) or (meta-evaluation* or meta evaluation*) or (rapid evidence assess*)). The search was limited to works published between 1996 and October 2009, journal articles, and English language articles. The Business Source Complete database was searched using the terms: 'realis$ review' OR 'realis$ synthesis' OR 'reali$ evaluation' OR 'meta-method' OR 'meta method' OR 'realis$ approach' OR 'meta-evaluation' ((rapid literature review) or (rapid systematic review) or (rapid scoping review)) OR ((rapid review) OR (rapid approach) OR (rapid synthesis)) OR ((meta-method* or meta method*) OR (meta-evaluation* or meta evaluation*) OR (rapid evidence assess*)). The search was limited to scholarly (peer reviewed) journal articles published between 1996 and October 2009. The journal *Evaluation *was also searched individually via the Sage website using the search term 'realist,' inclusive of dates between 1996 and October 2009. The Cochrane Methodology Registry was searched using the search term 'rapid review' and was also searched to examine the implications of various methodological streamlining approaches.

A thorough Internet search was conducted in July 2008 using *Grey matters: A search tool for evidence-based medicine *(Canadian Agency for Drugs and Technologies in Health [CADTH], 2008), using the search terms 'rapid review'; 'rapid approach'; 'rapid synthesis'; 'meta-method'; 'meta-evaluation'; 'rapid evidence assessment'; 'expedited review'; 'accelerated review' and 'realist review.' An updated internet search was conducted in November 2009. This Internet search included 55 health technology agencies, 12 health economic databases, 15 clinical practice guideline databases, six drug and device regulatory approval databases, six advisories and warnings databases, 14 free databases of published and unpublished literature, two health statistic databases, three open access journal databases, as well as two 'miscellaneous' internet searches. Efforts were made to identify any additional unpublished studies through contact and consultation with experts in June 2008 and November 2009. The reference lists of key relevant articles were hand searched and additional articles were identified.

### Criteria for selecting articles for this review

Two investigators independently reviewed titles and abstracts for relevance. All articles assessed as relevant were included for full text review for relevance (total = 70 articles). The criteria for inclusion were that the study publication date was 1995 or later, and the article concerned methods or examples of how to conduct a rapid review or addressed what may be lost in conducting a rapid review versus a traditional systematic review. An investigator and a research assistant then independently conducted full text reviews for relevance; any disagreement was resolved by discussion. The eligibility criteria were pilot tested for the first 10 articles to ensure consistent application by both reviewers. Reviewers were not blind to the name of the authors, institutions, journal of publication, and results when applying the eligibility criteria. Through full text review, 45 methodological articles were identified as relevant. As well, many examples of rapid reviews were identified.

## Results

**Figure 1:**
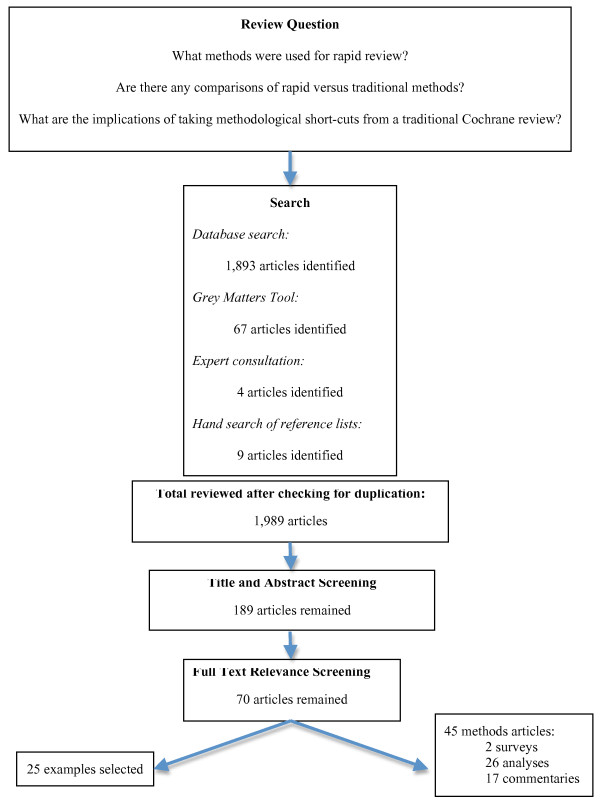
**Search Results**. Search process for articles included in this review.

Figure 1 outlines the number of articles involved in this review. The search process for published and unpublished literature resulted in the identification of 1,989 potentially relevant articles. After two reviewers independently screened these titles and abstracts 205 articles remained for full text screening. A total of 70 articles were identified as relevant for this review; 45 are methodological articles, while 25 are exemplars of the diverse rapid review methodological approaches that exist. All of the included articles and where they were located within the literature search (*i.e.*, grey, published, or consultation with experts) are summarized in Table 1.

**Table 1:** Summary of Included Studies and Sources of Studies: Included studies and where located within literature search

Author/Year	Literature Source:
Burls, 2004 [13]	CONSULTATION WITH EXPERTS
Butler *et al*., 2005 [23]	GREY
Cameron, 2007 [17]	GREY
Doust *et al*., 2005 [28]	PUBLISHED
Edwards *et al*., 2002 [33]	PUBLISHED
Egger *et al*., 2003 [40]	PUBLISHED
Egger and Smith, 1998 [22]	PUBLISHED
Eisenberg and Zarin, 2002 [19]	PUBLISHED
Elliott *et al*., 2006 [7]	GREY
Garces, 2006 [9]	GREY
Government Social Research (Ed.), 2007 [20]	CONSULTATION WITH EXPERTS
Hailey, 2007 [1]	PUBLISHED
Hailey *et al*., 2000 [45]	PUBLISHED
Helmer, 2001 [46]	GREY
Hopewell *et al*., 2007a [29]	PUBLISHED
Hopewell *et al*., 2007b [34]	PUBLISHED
Jadad *et al*., 1998 [21]	PUBLISHED
Juni, Altman, and Egger, 2001 [48]	PUBLISHED
Juni *et al*., 2002 [37]	PUBLISHED
Langham, Thompson, and Rowan, 1999 [30]	PUBLISHED
Lawson *et al*., 2005 [41]	PUBLISHED
Lehoux *et al*., 2004 [6]	PUBLISHED
McAuley *et al*., 2000 [35]	PUBLISHED
McManus *et al*., 1998 [31]	PUBLISHED
Moher *et al*., 1996 [43]	PUBLISHED
Moher *et al*., 1998 [47]	PUBLISHED
Moher *et al*., 2003 [39]	PUBLISHED
Moher *et al*., 2007 [49]	PUBLISHED
Moher and Altman, 2009 [10]	GREY
National Institute for Health and Clinical Excellence, 2009 [8]	GREY
Oliver *et al*., 2005 [50]	PUBLISHED
Oxman, Schunemann, and Fretheim, 2006a [3]	PUBLISHED
Oxman, Schunemann, and Fretheim, 2006b [5]	PUBLISHED
Page and Elliott, 2006 [53]	GREY
Parker, 2006 [66]	GREY
Pham *et al*., 2005 [42]	PUBLISHED
Royle and Milne, 2003 [25]	PUBLISHED
Royle and Waugh, 2003 [27]	PUBLISHED
Sampson *et al*., 2003 [26]	PUBLISHED
Savoie et al, 2003 [32]	PUBLISHED
Sterne, Gavaghan, and Egger, 2000 [36]	PUBLISHED
Topfer *et al*., 1999 [24]	PUBLISHED
Vickers *et al*., 1998 [44]	PUBLISHED
Watt *et al*., 2008a [4]	PUBLISHED
Watt *et al*., 2008b [18]	PUBLISHED

Although knowledge of rapid review techniques is expanding, limited methodological research exists and, overall, many rapid review reports lack transparency in terms of methods employed. Lehoux *et al*. conducted a study related to the knowledge and production of health technology assessments and reached similar conclusions [6]. Lehoux *et al*. acknowledge the need for short reports for policy and decision makers, yet also suggests a need for publishing these rapid reviews within peer-reviewed journals with greater emphasis on describing the methods used. Within this literature review, the majority of references provided examples of rapid reviews, with varying amounts of detail on the methods used. Some additional studies and methodological articles either discussed rapid review methodologies or addressed methodological implications of the streamlined steps used within some rapid reviews.

### Rapid methods used within examples of rapid reviews

#### Nomenclature

International rapid reviews vary widely in terms of the language used to describe these reviews, timeframes to complete them, content of the reviews, and methods. Various terms associated with rapid or accelerated methods for conducting reviews found within the literature include: 'rapid review,' 'rapid health technology assessment,' 'rapid response,' 'ultra rapid review,' 'rapid evidence assessment,' 'technotes,' 'succinct timely evaluated evidence review,' and 'rapid and systematic reviews.' These rapid reviews vary in the length of time taken to conduct literature reviews and synthesis, with timeframes ranging from one to nine months. Some reviews called themselves 'rapid,' yet used timeframes similar to those of traditional systematic reviews or were unclear about steps taken to accelerate their approach. Many studies failed to acknowledge the length of time taken to conduct the reviews. Some organizations that conduct rapid reviews have made available general guidelines about their rapid review products and processes. For example, the National Institute for Health and Clinical Excellence (NICE) has developed guidelines for rapid response products or health technology assessments that are usually completed within approximately nine months [7,8]. Garces briefly described the rapid review process used by the Canadian Agency for Drugs and Technologies in Health, stating that these reviews provide enhanced rigour beyond health technology inquiries, usually take four months to complete once the scope of review is defined, and follow a format similar to their full health technology assessments [9].

#### Methodological Approaches

Rapid reviews employ a variety of methodologies and vary in terms of the depth of description of methods used to make the processes rapid. Very few reviews explicitly address the questions of what was lost or what bias was introduced by using these methods. Numerous examples of rapid review methods were found; exemplars were chosen to demonstrate maximal variability in terms of methods used for rapid reviews found within this literature search (Table S1, Additional file 1,). We considered framing the various rapid review methods in the context of time taken to complete the syntheses; however, many did not report this information, and time required to conduct reviews is also dependent on staff availability. Instead, Table S1 Additional file 1 has been organized in terms of implications of methodological shortcuts taken, from minimal to significant levels of bias potentially introduced that would impact estimates of effectiveness as a consequence of the methodological approach. While we have suggested the implications of choosing the various methods, we acknowledge that the evidence, direction, and magnitude of any risk of bias cannot truly be assessed if methods have not been fully described [10]. Moreover, although a decision was made to structure the table based on potential for bias, part of this assessment is inevitably subjective because there is no way to quantify the relative impact of some methodological decisions (*e.g*., exclusion by study design versus failure to include grey literature).

Many reviews introduced restrictions at the literature-searching and retrieval stages. Several searches were truncated to include only readily accessible published literature, including limitations by language and date of publication, or by number of electronic databases searched. Others conducted systematic searches of published literature, yet limited searches of unpublished literature. One rapid review narrowed its search in terms of geographical context and setting (*i.e*., primary healthcare), to ensure that evidence could be readily applied to the context of interest [11]. Some acknowledged that their literature review and search term selection were not iterative processes, so some relevant references may have been missed [12]. Several others acknowledged restricted timeframes for articles to be retrieved and assessed, and limited ability to follow up with authors and industry contacts to clarify information presented [12-16]. Some rapid reviews streamlined systematic review methods at later stages in the process, including during title and abstract review, full text review, data extraction, and quality assessment phases.

#### Comparisons of rapid versus traditional methods

A review comparing rapid versus full systematic reviews found that overall conclusions did not vary greatly in cases where both rapid and full systematic reviews were conducted [17]. In terms of content, however, full reviews were more likely than rapid reviews to report clinical outcomes, economic factors, and social issues. Systematic reviews were also more likely to provide greater depth of information and detail in recommendations. Due to the various and variable differences between systematic and rapid reviews, it is suggested that rapid reviews may be useful to answer certain types of questions, but they are not viable alternatives to full reviews. Based on Cameron's inventory of current rapid review methods, it is also suggested that while standardization of rapid review methods may not be appropriate, it is important that transparency of methods be achieved [17]. Watt *et al*. found that although the scope of rapid reviews is limited, they can provide adequate advice for clinical and policy decisions [18]. Watt *et al*. also acknowledge that rapid reviews may not be appropriate for all healthcare or technology assessments. In a review of health technology assessments (HTAs) in the United States, Eisenberg and Zarin discussed increased pressure by Medicare to conduct assessments within shortened timeframes (approximately 45 days), while maintaining transparency and scientific rigour [19]. Eisenberg and Zarin identified a number of concerns associated with rapid HTAs, including: the complex nature of many questions; the scarcity of methodological and content knowledge for many rapid HTA topics; the challenges associated with synthesizing studies of lesser quality; and the need for methodological transparency to enhance scientific credibility of the rapid HTA process.

In a methodology discussion paper, Burls *et al*. also stated the need for transparency of methods used, particularly in the absence of standardized methods for thorough yet non-systematic literature searches [13]. The discussion paper also recommended minimum reporting standards related to rapid review methods. Oxman, Schunemann, and Fretheim recommended that rapid reviews should be explicit in terms of methods, limitations, and biases, but should also state the need for follow-up with a full systematic review [5].

Few articles explicitly summarized or focused on rapid review methodologies. Elliott *et al*. provided details about the rapid response process for NICE in the United Kingdom [7]. Updated and revised guidelines have recently been published by NICE [8]. Its rapid review process included: a six- to nine-month timeframe; needs assessment to provide clear understanding of the issue; an initially broad literature search to develop scope; consultation with key stakeholders to refine and focus the scope; guidance development over four months; and peer review or public consultation about results of the draft summary report [7]. *The Magenta Book: Guidance notes for policy evaluation and analysis*, by the Government Social Research Unit in England, discussed rapid evidence assessments that fall methodologically between health technology assessments and systematic reviews and are completed within two to three months [20]. These rapid reviews synthesize available evidence using 'fairly comprehensive' search strategies and sift out poor-quality evidence, but do not exhaustively search published and grey literature.

#### Implications of methods employed: Limitations and bias

##### Establishing Cochrane as the 'gold standard' in the continuum of rapid to systematic reviews

Within this literature review, a selection of articles addressed the implications of methodological techniques on bias. Jadad *et al*. conducted a comparison study of the methodological and reporting aspects of Cochrane reviews versus reviews found within paper-based literature [21]. This study found that Cochrane reviews were more likely than non-Cochrane reviews to be updated, and did not contain the language restrictions often found in non-Cochrane reviews. While no significant differences were found between these types of reviews in terms of sources of trials, frequency of heterogeneity testing or effect estimates, Jadad *et al*. suggested that Cochrane reviews are less prone to bias due to more explicit trial quality criteria, as well as inclusion and exclusion criteria [21].

#### Publication bias

Bias can be introduced in many ways through the methodological approach to study location and selection [22]. Butler *et al*. outlined methods used in rapid evidence assessments (REAs), and acknowledged that selection bias, publication bias, and language of publication bias may be introduced when using literature that is readily accessible to a researcher [23]. Within their REAs, exhaustive database searching, hand searching, and grey literature searching is not initially undertaken. Furthermore, it was suggested that the shortened timeframe associated with REAs increased risk of publication bias [24].

Topfer *et al*. compared literature searches within MEDLINE and EMBASE electronic databases and found that the greatest yield of relevant resources came from combined searches of the databases, because each identified resources not found in the other [24]. Topfer *et al*. acknowledged that better search strategies are partnered with increased time and cost for reviewers. While Royle and Milne also found that additional database searching produced additional trials, this remained only a small percentage of overall number of trials [25].

Sampson *et al*. found that searching MEDLINE but not EMBASE has the potential to impact meta-analysis effect size estimates, suggesting a potential for database bias [26]. Royle and Waugh compared the cost-effectiveness of various literature retrieval strategies and found diminishing marginal returns with increased database searching [27]. Instead, Royle and Waugh recommended that, when timeframes are restricted, hand searching of relevant reference lists and consultation with experts about missed articles may be more effective than exhaustive database searching. Oxman, Schunemann, and Fretheim supported this recommendation and also suggested that when conducting rapid assessments with limited resources, priority should be placed on quality assessment over extensive literature searching. In addition, contacting experts and hand searching reference lists should be given priority over additional database searching [3].

Doust *et al*. compared sensitivity and precision of search strategies, comparing use of bibliographic databases with hand searching for references [28]. This study highlighted the potential for increased accuracy but decreased practicality in hand searching a large number of journals. Doust *et al*. recommended using 'snowballing' techniques, and also having two reviewers screen citation lists to maximize sensitivity of bibliographic searching. Hopewell *et al*. compared hand searching versus electronic searching and found that a combination of these approaches provide the most comprehensive results when searching published literature [29]. Hopewell *et al*. found that hand searching provided greater search yields than electronic searching alone, and suggests this is likely related to indexing of terms within the databases [29]. Langham, Thompson, and Rowan compared hand searching versus MEDLINE searching in terms of emergency medicine literature, with similar conclusions: hand searching is better than electronic searching, but a dual approach to literature searching should be employed [30]. Accuracy of hand searching is, however, dependent upon the knowledge and expertise of those conducting the searches.

McManus *et al*. reviewed the importance of contacting experts in literature searching, indicating that electronic searching may only locate one-half of relevant studies, and that 24% of relevant studies may be missed by not contacting experts [31]. Contacting experts is particularly important in fields lacking well-defined specialist literature, because hand searching is often focused on such specialist literature. Savoie *et al*. studied sensitivity and precision of extended search methods, and found that searching beyond electronic databases, with specialized databases and trial registries, was most effective for identifying relevant randomized controlled trials [32]. In addition, Edwards *et al*. examined the accuracy and reliability of reviewers in screening records, and found that while a single reviewer is likely to identify the majority of relevant records, having a second reviewer maximizes inclusion and can increase the records identified by an average of 9% [33].

#### Small and unpublished study effects

A few studies addressed the impact of grey literature on treatment effect within meta-analyses. A Cochrane review of the impact of grey literature in meta-analyses of randomized controlled trials found that the inclusion of grey literature decreased publication bias and provided more conservative treatment effects than when grey literature was excluded [29]. Hopewell *et al*. had results consistent with this; published trials were typically larger and showed greater treatment effects than those found within grey literature [34]. McAuley *et al*. found that exclusion of grey literature could lead to inflated effectiveness estimates, and suggested that meta-analyses should seek to include all grey and unpublished reports that meet study inclusion criteria [35]. In contrast, Sterne, Gavaghan, and Egger examined the impact of small study effects on meta-analyses, and found that inclusion of smaller studies may increase treatment effects and introduce bias due to potentially lower methodological quality [36].

#### Language of publication bias

Other studies addressed the impact of other languages on treatment effects and conclusions in meta-analyses [37,38]. Juni *et al*. found that inclusion of non-English studies typically involved greater efforts to locate, as well as cost and time to translate, but exclusion led to more conservative treatment effect estimates [37]. Juni *et al*. also concluded that the need to include non-English studies may depend on the topic of the review, and whether relevant studies within the specialty literature are predominately published in English. In contrast to these findings, Moher *et al*. found that language restricted meta-analyses did not differ significantly in intervention effectiveness estimates when compared to language inclusive meta-analyses [38,39].

Egger *et al*. suggest that if the content area of a review is housed primarily within published literature, then a review based on a search of English language-restricted studies will likely produce similar results to those based on those that do not have language restrictions [40]. Lawson *et al*. found that systematic reviews that did not restrict searches by language tended to be more comprehensive in their searches and inclusion of relevant literature [41]. They did, however, find that systematic review results can be influenced by restricting languages if their language of publication is associated with study quality [42]. The influence of language is also dependent upon whether the review is based on conventional medicine or complementary and alternative medicine [42]. It has also been suggested that depending on the content area of the planned review, investigators need to consider the literature search and the level of comprehensiveness of searching necessary [40]. For example, the methodological quality of harder-to-find studies also needs to be considered, as they may be of lower methodological quality and actually increase bias by their inclusion [40]. In contrast, specific to other-language trials, Moher *et al*. found no difference in trial quality and reporting among English and other language trials, and suggest that inclusion of other languages can increase precision and reduce language of publication bias [43]. An additional consideration beyond language is country or location of study publication. Vickers *et al*. found that some countries publish higher proportions of positive results (*i.e*. publication bias), which may have implications for rapid review results if a search is limited by publication location [44].

## Discussion

Within the rapid review studies, some authors acknowledged that accelerating the data extraction process might lead to missing some relevant information. Some also acknowledged that publication bias might be introduced due to shortened timeframes for literature searching and article retrieval. Watt *et al*. conducted a review of current methods and practice in HTAs, and suggests that due to the limitations associated with rapid reviews, conclusions may be less able to be generalized and may provide less certainty than those of traditional systematic reviews [4]. Rapid reviews with shorter timeframes (one to three months) were often less systematic in their search for evidence than those with longer timeframes (three to six months). Watt *et al*. suggested that this might lead to uncertainty around the conclusions drawn and inability to answer certain types of questions (*e.g*., economic analyses).

Hailey also found that the nature of the advice provided within rapid reviews was typically limited to questions related to efficacy or effectiveness [45]. Burls *et al*. highlighted steps that should be taken to ensure that the rapid guidance produced is authoritative, including consulting with key stakeholders in the process and preparation of reports and decision-making about the use of the technologies by those external to the review process [13]. As such, the process of producing the scientific evidence and the subsequent policy development should also be separate processes. Furthermore, a well-formulated question and well-defined context is imperative [13].

Due to the limitations in drawing conclusions and ability to answer questions, rapid reviews should only be viewed as interim guidance until more systematic reviews can be conducted [4,45]. While rapid reviews should not be seen as alternates to systematic reviews, Cameron suggests that exhaustive data searching may not greatly impact final conclusions and recommendations of a review [17]. In contrast, Helmer compared MEDLINE searching versus extended searching that included specialist databases, hand searching, reference list review, and personal communication with experts, and found that systematic searching increases the number of studies found and decreases bias [46]. Helmer suggests that the likelihood of extended searching impacting the number of items retrieved may depend on the content area and whether content is likely to be found in mainstream databases. In comparing mainstream versus extended search strategies, no difference in quality of studies was found [46].

In defining parameters for rapid reviews, Burls *et al*. suggested that search efforts should be focused on those resources that are most likely to affect the outcome of the evaluation [13]. Limitations of rapid reviews need to be weighed against the additional cost and time associated with systematic searching, inclusion of non-English studies (including translation), and searching of grey literature. Some literature has identified that inclusion of non-English studies can impact treatment effects; the literature is unclear, however, about the nature of this impact [37]. Butler *et al*. suggested that all rapid evidence assessments should carry the caveat that conclusions may be subject to change and/or revision once a more systematic review has been completed [23]. *The Magenta Book *also suggests that all rapid reviews should carry a similar qualifying statement [20].

While limiting the literature search strategy is a common strategy for rapid reviews, it is not the only approach to methodological streamlining. One of the concerns identified, in examining the methods of the exemplars, was a lack of quality assessment in some of these reviews. If the quality assessment process is eliminated or not articulated, this has much more substantial implications for the results of the review [3]. A study by Moher *et al*. found that trial quality can significantly impact benefit effect sizes [47]. Juni, Altman, and Egger also suggest that failure to conduct quality assessments of primary studies can distort the results of a review [48]. If quality assessment is not part of the rapid review approach, there are clearly substantial limitations associated with the literature synthesis process and the utility of the results. This also raises the question of whether the review should be considered a rapid review.

## Limitations

Although numerous examples of rapid or accelerated reviews were found, many of these articles were not explicit about the methodology employed--specifically, where their process was streamlined. In addition, very few discussed limitations associated with or bias introduced by the streamlining process. However, several studies acknowledged that their report is not a comprehensive systematic review, and should be viewed as interim guidance that should be followed up by a thorough review. While several types of bias have been discussed within the paper, the list is not exhaustive, and there may be other potential types of bias that may impact the results of rapid reviews that have not been addressed.

Furthermore, despite the above described differences in quality between the spectrum of rapid review approaches and systematic reviews, in a study of reporting characteristics of systematic reviews, Moher *et al*. found that the quality of systematic reviews themselves are inconsistent, thereby blurring lines further between systematic review and rapid review methods [49]. Moher *et al*. recommended evidence-based reporting guidelines, which would be beneficial to both systematic reviews and rapid review products.

## Summary

This review examined rapid review methods and the implications of streamlining traditional systematic review processes. Seventy relevant articles were included in this review. Forty-five were methodological articles, while the remaining articles were examples of rapid review studies that varied widely in the methods used. While one consistent methodological approach may not be optimal or appropriate [50], it is important that future rapid reviews are transparent both in terms of methods used and limitations or biases introduced by these approaches. Further research comparing full systematic reviews with rapid reviews will enhance understanding of the limitations of these methods.

## Competing interests

The authors declare that they have no competing interests.

## Authors' contributions

RLG carried out the grey literature search, full text screening, data extraction and analysis, and drafted the manuscript. DC conceived of the study, participated in the design of the study, title and abstract screening, full text screening, and contributed to the manuscript drafts. HT participated in the study design, title and abstract screening, and contributed to the manuscript drafts. All authors read and approved the final manuscript.

## Supplementary Material

Additional file 1 **Table S1 - Summary of Included Studies - Exemplars and rapid review method employed**. Rapid review exemplars and implications of methodological shortcuts [51-71]. Click here for file
