# Supplementary material for: Expediting systematic reviews: methods and implications of rapid reviews
Source: Implement Sci. 2010 Jul 19;5:56. doi: 10.1186/1748-5908-5-56 (PMC2914085; doi:10.1186/1748-5908-5-56)
Supplement: Additional file 1 — Table S1 - Summary of Included Studies - Exemplars and rapid review method employed. Rapid review exemplars and implications of methodological shortcuts [51-71]. [file 1748-5908-5-56-S1.DOC]

**Table S1**. Summary of Included Studies – Exemplars and rapid review method employed

| **Author/Year** | **Method Streamlined** | **Limitations identified by author(s)^1^** | **Located: Published or grey literature** |  |
| --- | --- | --- | --- | --- |
| **implications on effectiveness: minimal** | | | |  |
| McRobbie, 2006 [51] | Narrow search criteria, date restrictions through consultation with experts. Some search for grey literature but not systematic. One reviewer conducted title and abstract review. | Limitations in terms of ability to provide details about sub-analyses | Grey |  |
| Clark et al., 2003 [52] | Narrow search criteria through consultation with experts. Used expert consultation throughout process to ensure clinical relevance and increase sensitivity of search strategies. |  | Grey |  |
| Page and Elliot, 2006 [53] | Consensus methods related to scoping review |  | Grey |  |
| Singh, 2006 [15] | One reviewer conducted data extraction. |  | Grey |  |
| Low et al., 2006 [54] | Quality assessment conducted by two reviewers for systematic reviews, controlled trials, before and after studies, time trend studies. Data extracted from cross-sectional studies, other non-controlled observational studies and qualitative studies by one reviewer. |  | Grey |  |
| O’Meara et al., 2001 [55] | Some language restrictions. Did not discuss consultation with experts for additional relevant sources. Restricted study design to randomized controlled trials. |  | Published |  |
| Lewis et al., 2001 [56] | Restricted study design to randomized controlled trials and non-randomized controlled trials with full economic evaluation. |  | Published |  |
| Cummins et al., 2001 [57] | Restricted study design to randomized controlled trials and systematic reviews. |  | Grey |  |
| Facey et al., 2007 [58] | English articles only (therefore called it an ‘ultra rapid review). |  | Published |  |
| **Implications on effectiveness: Moderate** | | | |  |
| Butler, 2004 [12] | Restricted study design to experimental or quasi-experimental designs. List of search terms developed; search terms were ‘one-off’ (not iterative). Discusses that exhaustive database searching and hand searching of journals or grey literature were not immediately conducted. List of search terms developed; search terms were ‘one-off’ (not iterative). | Only included published studies, restricted publication dates, used strict cut-off date for article retrieval, study design, focused on strength of evidence not program effectiveness. Discuss potential for selection and/or publication bias. Acknowledge narrow timeframe for arrival of articles. Describes ‘rapid evidence assessment’ as interim assessment of evidence. Timeframe: less than six months. | Grey |  |
| Singh, 2005 [59] | Narrow search criteria through consultation with experts. One reviewer conducted title and abstract review for inclusion/exclusion. | States it is not an exhaustive review but rather a general summary of trends related to planned components of program. Only ‘readily available’ published or unpublished documents included. | Grey |  |
| Vlayen et al., 2006 [60] | Narrow search criteria through consultation with experts. Search for existing health technology assessments, systematic reviews, and randomized controlled trials; also date restrictions. Quality assessment conducted by one reviewer. | Search for published literature only. Patient issues, ethical issues and organizational issues not addresses | Grey |  |
| Burls et al., 2002 [14] | Narrow search criteria (scope of questions) through consultation with experts. No follow up with missing data from pharmaceutical industry. | Discuss timeframe of four to six weeks to read and extract data from industry submissions, therefore relevant information may have been overlooked. | Grey |  |
| Muthu, 2001 [61] | Only published literature plus hand searching of references, consultation with experts. One reviewer conducted title and abstract review. Used peer review of draft manuscript. | Acknowledge that users of report should check for later evidence that may alter conclusions. | Grey |  |
| Birmingham and Black County Strategic Health Authority, 2008 [62] | Some grey literature searching but not systematic. | Acknowledges that review did not set out to be ‘exhaustive’ but rather has the goal of providing overview of major trends.  Provides caveats related to interpretation, including:   1. If review suggests that intervention does not impact the outcomes of interest, that does not mean that it is without merit; it may have an impact on other outcomes. 2. Limitations of limited evidence—also does not mean initiative has no impact.   Context of interventions may limit application of findings to other regions/ countries. | Grey |  |
| Adi, Bayliss, and Taylor, 2004 [63] | No discussion of search for grey literature aside from focused search of health technology assessment websites. | Discussed limitations of time frame for West Midlands Health and Technology Assessment Collaboration reviews (usually three to six months). | Grey |  |
| Joliffe and Farrington, 2007 [64] | Focused internet search. (Contacted lead researchers, electronic database searching and focused internet searching were used.) | Discuss publication bias since difficult to obtain material and non-English studies were not included. Found that studies with high methodological quality showed smaller effect sizes, whereas studies of average quality showed the greatest effect size. Smaller studies showed a greater impact than larger studies, which may be due to better quality control. | Grey |  |
| Bryant and Gray, 2006 [11] | Narrowed search in terms of geographical context and setting. |  | Published |  |
| Middleton, Simpson and Maddern, 2003 [65] | Database searching included: MEDLINE, Pre-MEDLINE, Cochrane. Inclusion/exclusion method used not discussed. Restricted study design to randomized controlled trials. |  | Grey |  |
| **Implications on effectiveness: Moderate** | | | | |
| Singh and Ham, 2006 [16] | Only published literature used. Review of literature and feedback completed in a three-week time frame. | Only readily available published research is included. Identify that it is not an exhaustive review. Discusses limitations in quality and scope of evidence. Acknowledges limitations of data synthesis process. Caveats: included literature that was readily accessible in a three-week period; limitations related to context and ‘usual cure’ that differs across countries. | Grey |  |
| Parker, 2006 [66] | Rapid review plus ‘experience review’. Methodology for rapid review not explicit. Literature review included is readily available published research. | Review does not attempt to provide a complete national picture of programs and projects. Instead it provides a range of examples. | Grey |  |
| Coomber et al., 2004 [67] | Sought input from experts for article inclusion. Selective focus on databases (*i.e.*, compiled list of databases). No formal quality assessment. | Acknowledge narrow timeframe for arrival of articles. | Grey |  |
| London Health Commission, 2008 [68] | Some grey literature searching but not systematic. Process for inclusion/exclusion, data extraction, and quality assessment not explicitly reported. | Stress that not all evidence is of comparable quality. Articulate what is within and outside of scope of work. | Grey |  |
| Foerster, Murtagh, and Fiander, 2007 [69] | English articles only, date restrictions. One reviewer conducted full text review for inclusion/exclusion. No formal quality assessment. | Acknowledge limited literature scope. Discusses limitations of literature found (*i.e.*, methodologically weak); limited time for production of report; limited in scope and rigor (*i.e.*, no formal quality assessment). | Grey |  |
| Australian Safety Efficacy Register of New Interventional Procedures Surgical, 2003 [70] | Did not discuss consultation with experts. Data extraction completed but number of reviewers/process not discussed. No formal quality assessment process. |  | Grey |  |
| World Health Organization, 2006 [71] | Narrow search criteria through consultation with experts. Panel of experts reviewed evidence, developed process. Evidence profiles prepared, revised and draft guidelines developed. Draft manuscript prepared (10 days). | Used very focused search scope, reference lists were searched, used expert review of lists. |  |  |

^1^Where limitations not identified (column 3), authors did not explicitly state limitations/implications of rapid review methodology employed.
